# The effect of different modes of hypobaric hypoxia on the expression of transcription factor pCREB and pro-survival proteins BDNF and BCL-2 in rat neocortex and hippocampus

**DOI:** 10.1186/2193-1801-4-S1-L27

**Published:** 2015-06-12

**Authors:** Anna Churilova, Michail Samoilov

**Affiliations:** Pavlov Institute of Physiology of Rissian Academy of Sciences, Russian Federation, Russia

**Keywords:** Hypoxic preconditioning, brain hypoxic tolerance, pCREB

Severe hypobaric hypoxia (180 mm Hg) is a harmful stimulus that induces structural and functional injures of susceptible brain neurons in the neocortex and hippocampus. In contrast, moderate hypobaric hypoxia (360 mm Hg) activates endogenous cellular defensive mechanisms. The rate of neuroprotection afforded by the mild hypoxia depends on the quantity of hypoxic sessions. In particular, preconditioning (pre-exposure) by three trials of mild hypoxia protects from deleterious effects of subsequent severe hypoxia whereas preconditioning with one mild hypoxic trial does not. The molecular mechanisms induced by mild hypoxic preconditioning are unclear. Pro-survival proteins, such as neurotrophic factor BDNF and anti-apoptotic factor Bcl-2 are supposed to be involved in this process. In the present study, the effects of three-trial and one-trial hypoxic preconditioning on the expression of pro-survival proteins BDNF and Bcl-2 as well as their up-stream activator pCREB, have been studied in the neocortex and hippocampus of rats. As revealed by quantitative immunocytochemistry, the severe hypobaric hypoxia didn’t affect or down-regulated the neuronal levels of pCREB, BDNF and Bcl-2 at 3-24 h after the exposure. The one-trial preconditioning did not change this effect of severe hypoxia. In contrast, preconditioning by three trials of mild hypoxia (360 Torr, 2h, 24 h intervals, 3 times) significantly enhanced the pCREB, BDNF and Bcl-2 neuronal expression in response to severe hypoxic challenge. Three-trial mild hypoxia alone also up-regulated the expression of molecular factors examined in the neocortex and hippocampus at 24 h whereas one trial of the mild hypoxia did not. The results of the present study indicate that development of the neuronal hypoxic tolerance induced by the three-trial, in contrast to one-trial, mild hypoxic preconditioning is apparently largely associated with the activation of CREB, as well as BDNF and Bcl-2 overexpression.

